# Lateral interbody fusion without intraoperative neuromonitoring in addition to posterior instrumented fusion in geriatric patients: A single center consecutive series of 108 surgeries

**DOI:** 10.1016/j.bas.2023.101782

**Published:** 2023-07-11

**Authors:** Lukas Bobinski, Per Liv, Bernhard Meyer, Sandro M. Krieg

**Affiliations:** a Department of Neurosurgery, Klinikum rechts der Isar, Technische Universität München, Munich, Germany; b Umeå University Hospital, Spine Unit, Umeå, Sweden; c Section of Sustainable Health, Department of Public Health and Clinical Medicine, Umeå University, Umeå, Sweden

**Keywords:** Anterior spine surgery, Lateral thoracic interbody fusion, Lateral lumbar interbody fusion, IONM, Neuromonitoring

## Abstract

**Introduction:**

Lateral lumbar interbody fusion (LLIF) and lateral thoracic interbody fusion (LTIF), supported by intraoperative neuromonitoring (IONM), gained popularity as a mini-invasive alternatives for standard interbody fusion. The objective of this study was to investigate the clinical outcome in a large elderly patient cohort who underwent LTIF/LLIF without IONM.

**Methods:**

This retrospective, single-center study enrolled elderly patients (≥70 years old) operated during the period from 2010 to 2016. Anterior lumbar interbody fusion (ALIF) in the L5/S1 segment was excluded from the analysis.

**Results:**

The study enrolled 108 patients (63 males, 58.3%) with a mean age of 76.5 ​y/o. The mean follow-up was 14.4 ​± ​11.3 months. The mean time of the surgery was 92 ​± ​34.2 ​min. The mean blood loss was 62.2 ​ml. There were no vascular or visceral surgical complications. 39 medical complications were encountered in 24 (22%) patients. Less than 5% of patients presented with a new onset of motor weakness and less than 2% of the patients developed a new sensory deficit at the discharge. 46% of patients were lost in follow-up at 12 months.

**Conclusions:**

IONM is not mandatory for LLIF/LTIF surgery in geriatric patients and has a low frequency of approach-related complications as well as neurological deterioration. Our results are comparable to the available literature. Regardless of the utilization of these mini-invasive, anterior approaches, in patients of advanced aged, the risk for major medical complications is high and is responsible for contributing to prolonged hospitalization.

## Highlights

• This study investigates an elderly patient cohort undergoing lateral interbody fusion without IONM.
• The study enrolled 108 patients (63 males, 58.3%) with a mean age of 76.5 ​y/o.
• 39 medical complications were encountered in 24 (22%) patients.
• Less than 5% of patients presented with a new onset of motor weakness.
• Less than 2% of the patients developed a new sensory deficit at the discharge.

## 1 Introduction

A growing number of geriatric patients requiring spine surgery due to degenerative spine conditions (Cooper et al., 2016; Benz et al., 2001; Deyo et al., 1992). Indeed, the number of instrumented lumbar surgeries is on the rise with spinal stenosis being the most common indication for surgery in patients older than 65 years (Deyo, 2010). Furthermore, the elderly present with as high as 68% prevalence for the adult spinal deformity (ASD), when compared to the general population (Schwab et al., 2005). These factors impose that spine surgeries should be planned accordingly to patients' age, to offer applicable surgical solutions to achieve good surgical results without unnecessary elevation of peri- and postoperative risks (Fehlings et al., 2015).

The lateral lumbar interbody fusion (LLIF) approach and lateral interbody thoracic approach (LTIF) were designed to minimize perioperative bleeding, decrease complication rate, and shorten hospital stay by providing superior discectomy, fusion and a large footprint cage at the same time (Ozgur et al., 2006). However, it carries the potential risk of the injury of the lumbar plexus within the psoas muscle, sympathetic chain as well as the intra-abdominal organs and major vessels (Czerwein et al., 2011; Wood et al., 2010; Wang and Mummaneni, 2010). Most authors agree that real-time intraoperative neuromonitoring (IONM) should be a mandatory part of the procedure in order to minimize the risks of injury to the lumbar plexus during the approach and manipulation of the psoas muscle (Tohmeh et al., 2011; Knight et al., 2009; Rodgers et al., 2011). Yet, other works, including our own, provide a different and more anatomy-oriented perspective (Lee et al., 2018; Krieg et al., 2019). Thus, IONM is neither the default procedure in LLIF nor is there any consensus yet, on how to avoid the especially frequent sensory deficits or dysesthesias after LLIF, for which MEP monitoring – as frequently used – does not provide any protection. Larger analyses even show IONM to be not protective at all (Nie et al., 2022). More data on the topic is therefore needed.

Our retrospective, single-center study investigates the incidence of complications and clinical outcomes in patients treated with minimally invasive LLIF without IONM as a previous study of our group did for the general population (Krieg et al., 2019). The goal of this study was to investigate the clinical outcome, pain, and complication rate in geriatric patients operated with LLIF and/or LTIF without IONM.

## 2 Materials and methods

### 2.1 Ethics

The study was approved by the local ethics committee (registration number: 159/16S). The analysis of data for this study was conducted in accordance with the Declaration of Helsinki.

### 2.2 Study design

The study enrolled elderly patients (≥70 years old) selected from a previously investigated cohort. 108 patients (63 males and 45 females) were included. The mean age at the time of surgery was 76.5 ​± ​4.4 years. All the patients were operated on using the LLIF approach in some cases combined with lateral thoracic interbody fusion (LTIF) at the department, between March 2010 and December 2016. All surgeries were performed without IONM support. Clinical and demographic data were extracted from the medical records. The diagnosis leading to the surgery included: spinal instability in 38 (35%) patients, pseudoarthrosis in 13 (12%) patients, discitis in 26 (24%) patients, ASD 25 (23%) patients, and fracture in 6 (6%) patients. 97 (97%) patients scheduled for LLIF/LTIF were operated on with posterior instrumentation the days before. 104 (96%) received also posterior decompression prior to LLIF/LTIF.

Approaches to L5/S1 disk space were excluded from the analysis. This segment can be easily accessed by the anterior lumbar interbody fusion (ALIF) technique, which does not require psoas manipulation and therefore rarely leads to neurological deterioration.

Preoperatively 75 patients (70%) presented with sciatica prior to the anterior surgery; 40 (37%) presented with paresis; and 17 (16%) presented with a sensory deficit.

To evaluate the risks, we decided to focus on three categories:

- complications directly related to the surgical approach
- postoperative neurological deterioration due to manipulation of the psoas muscle and lumbar plexus
- medical complications after the surgery

### 2.3 Surgery

Patients were positioned in a lateral position on the right side with the left side being exposed for surgical access. After fluoroscopic confirmation of aimed levels, lateral retroperitoneal or transthoracic dissection was performed to expose the lateral surface of the spine. Clydesdale® (Medtronic Sofamor Danek®) or Synframe® (Depuy Synthes®) retractor systems were used for access. IONM surveillance was not used in any of the surgeries. The meticulous dissection was carried out in a slightly oblique fashion, just to the edge of the psoas muscle, which was then gently mobilized and retracted in order to gain access to the disc space at zones I and II according to Uribe et al. (2010). After discectomy and the preparation of the endplates, a polyetheretherketone (PEEK) cage was implanted across the endplates. Demineralized bone matrix (DBM) was used in 81% of cases. Autologous bone harvesting was not performed in any of the cases.

To gain access to LTIF (lateral transthoracic interbody fusion) partial resection of one rib was carried out with the following short deflation of the lung. Diaphragm was incised and dissected only to get insight to the segments in the thoracolumbar junction (Th11/Th12, Th12/L1 levels). The disc space preparation was carried out in the same fashion as the retroperitoneal approach.

### 2.4 Statistics

Depending on whether the evaluated data are continuous or non-continuous the data are presented as the mean ​± ​standard deviation (SD) or the median and range. The neurological outcomes at discharge and follow-up (3 and 12 months) are presented with a confidence interval (CI). The initial analysis was performed partially via GraphPad Prism 6.04, La Jolla, CA, USA. Final analysis was performed using R statistic program version 4.1.1. The statistical analysis of association between medical complications as well as deterioration in neurologic status age, gender, specific operated levels, perioperative bleeding and time of the surgery were performed using Pearson's Chi-squared test and Kruskal-Wallis rank sum test respectively.

A univariate analysis was performed to investigate whether specific factors influenced motor deficit, sensory deficit, and leg pain at discharge, three months and one year postop: age group (70–74, 75–79, 80+), the operation time, blood loss the number of levels operated during one surgery and the preoperative status of the respective deficit. A P value ​< ​0.05 was statistically significant.

## 3 Results

### 3.1 General

All enrolled patients were followed both clinically and radiologically. We carefully reviewed all available medical records, particularly the neurological examinations before surgery, at discharge, and at 3 and 12 months of follow-up. Totally, 219 segments were operated by the anterior approach (2.02 levels/patient). Mean fused levels after the previous surgery were 5 (range 1–11). The mean operation time was 92 ​± ​34.2 ​min. The mean perioperative blood loss was 62.2 ​± ​57.8 ​ml. See details in Table.1.

**Table 1:** General characteristics General characteristics of investigated cohort. Number of patients, operated levels, mean blood loss and surgical time.

	Overall (N ​= ​108)
**Sex**	
Female	45 (41.7%)
Male	63 (58.3%)
**Age at surgery**	
Mean (SD)	76.5 (4.4)
**Instrumented segments**	
**T10/11**	5 (4.6%)

**T11/12**	12 (11.1%)

**T12/L1**	18 (16.7%)

**L1/2**	37 (34.3%)

**L2/3**	72 (66.7%)

**L3/4**	69 (63.9%)

**L4/5**	6 (5.6%)

**Number of fused levels in general**	
Median	5.0 (4.0, 7.0)
Range	1.0–11.0
**Blood loss (mL)**	
Mean (SD)	62.2 (57.8)
**Operation time (min)**	
Mean (SD)	92.0 (34.2)

Statistic significant correlation (p ​< ​0.05) was found between the pre-surgery status of all respective deficits in all models except for leg pain at maximal follow-up. Furthermore, the time of surgery demonstrated p-value close to significant in regard to the postoperative sensory deficit at discharge (0.06) and significant at 12 months (0.048) (Kruskal-Wallis rank sum test). We did not find any statistically significant correlation between remaining factors and postoperative deterioration. See details in Table 6, Table 7, Table 8.

### 3.2 Status at discharge

The data were obtained from all 108 patients enrolled in the study. 11 (10.2%) patients presented with improved and 5 (4.6%) with deteriorated muscle strength in comparison with their preoperative status; 92 patients (85.2%) presented with the unchanged status. Regarding sensory deficits, 5 patients (4.6%) presented with improved and 2 (2%) with deteriorated sensory deficits, whereas the vast majority 101 (93.4%) were unchanged. In terms of leg pain, 55 patients (51%) showed improvement, 5 (4.6%) complained about worsening, and 48 (44.4%) were unchanged compared with preoperative neurological status. See details in Table 2.

**Table 2:** Neurological status at discharge Detailed description of the neurological status (leg pain, sensory and motor strength) after surgery at the discharge.

Status at discharge	Leg pain	Motor deficit	Sensory deficit
		% [95%-CI] (N)	
**Improved**	0.509 [0.411,0.607] 55	0.102 [0.052,0.175] 11	0.046 [0.015,0.105] 5
**Unchanged**	0.444 [0.349,0.543] 48	0.852 [0.771,0.913] 92	0.935 [0.871,0.974] 101
**Deteriorated**	0.046 [0.015,0.105] 5	0.046 [0.015,0.105] 5	0.019 [0.002,0.065] 2
**Number of patients**	108	108	108

### 3.3 Postoperative follow-up at 3 months

The mean time between surgery and the first postoperative follow-up was 2.9 ​± ​1.7 months (range: 1–11 months). The data were obtained from 72 patients at that time. We found a persistent motor weakness in 4 patients (5.6%) and 5 patients (7%) still presented with persistent sensory deficit. Leg pain worse than before surgery was reported by 2 patients (3%). See details in Table 3.

**Table 3:** Neurological status at 3 months follow-up Detailed description of the neurological status (leg pain, sensory and motor strength) at the 3 months follow-up.

Status at first follow-up	Leg pain	Motor deficit	Sensory deficit
		% [95%-CI] (N)	
**Improved**	0.543 [0.419,0.663] 38	0.125 [0.059,0.224] 9	0.070 [0.023,0.157] 5
**Unchanged**	0.429 [0.311,0.553] 30	0.819 [0.711,0.900] 59	0.859 [0.756,0.930] 61
**Deteriorated**	0.029 [0.003,0.099] 2	0.056 [0.015,0.136] 4	0.070 [0.023,0.157] 5
**Number of patients**	70	72	71

### 3.4 Postoperative follow-up at 12 months

The mean time to planned one-year follow-up was 13.8 ​± ​10.7 months (range: 2–41 months). We noted a significant dropout, thus the data were obtained from 50 patients. The persistent motor deficit was found in 8 (16%) and sensory deficit in 5 (10%) examined patients, respectively. Leg pain was reported by 5 patients (10%). See details in Table 4.

**Table 4:** Neurological status at one year follow-up Detailed description of the neurological status (leg pain, sensory and motor strength) at the 1 year follow-up.

Status at first follow-up	Leg pain	Motor deficit	Sensory deficit
		% [95%-CI] (N)	
**Improved**	0.604 [0.453,0.742] 29	0.098 [0.033,0.214] 5	0.080 [0.022,0.192] 4
**Unchanged**	0.292 [0.170,0.441] 14	0.745 [0.604,0.857] 38	0.820 [0.686,0.914] 41
**Deteriorated**	0.104 [0.035,0.227] 5	0.157 [0.070,0.286] 8	0.100 [0.033,0.218] 5
**Number of patients**	48	51	50

### 3.5 Complications

There was no reported peri- or postoperative vascular or visceral complications. 24 (22%) patients developed medical 39 complications. 22 (56.4%) were classified as major and 17 (43.5%) as minor in accordance to the definition in the literature (Lebude et al., 2010). Details regarding the type and quantity of medical complications are depicted in Table 5.

**Table 5:** Medical complications Detailed description of number and type of medical complications.

Number of patients with medical complications	24/108 (22%)
***Number of complication (total)***	**39**
***Type of major complication****:*	**22 (56,4%)**
Pneumonia	8 (20,5%)
Anemia	1 (2,5%)
Pulmonary Embolism (PE)	5 (12,8%)
Multiple organ failure	2 (5%)
Kidney insufficiency	2 (5%)
Respiratory insufficiency	1 (2,5%)
Delirium	1 (2,5%)
*TIA*	1 (2,5%)
*Endocarditis*	1 (2,5%)
***Type of minor complications:***	**17 (43,5%)**
Deep Venous Thrombosis (DVT)	2 (5%)
Pleural effusion	4 (10%)
Urinary Tract Infection (UTI)	4 (10%)
Others^a^	7 (18%)

a *Anticholinergic syndrome, S. Epidermidis in the blood samples, Clostridium infection, Keratitis, Thrombocytopenia, Atrial Fibrillation (AF)*.

**Table 6:** Accumulated data from univariable analysis (pre-existing leg pain).

	No pain (N ​= ​66)	Pain (N ​= ​42)	p value
**Leg pain before surgery (discharge)**			< 0.001^a^
No leg pain pre-surgery	30 (45.5%)	3 (7.1%)	
Leg pain pre-surgery	36 (54.5%)	39 (92.9%)	
**Leg pain before surgery (3 month)**			0.042^a^
No leg pain pre-surgery	16 (32.7%)	2 (9.5%)	
Leg pain pre-surgery	33 (67.3%)	19 (90.5%)	
**Leg pain before surgery (1 year)**			0.851^a^
No leg pain pre-surgery	9 (25.7%)	3 (23.1%)	
Leg pain pre-surgery	26 (74.3%)	10 76.9%)	

a Pearson's Chi-squared test.

**Table 7:** Accumulated data of univariable analysis (pre-existing sensory deficit).

	No sensory deficit (N ​= ​94)	Sensory deficit (N ​= ​14)	p value
**Sensory deficit before surgery (discharge)**			<0.001^a^
No sensory deficit pre-surgery	89 (94.7%)	2 (14.3%)	
Sensory deficit pre-surgery	5 (5.3%)	12 (85.7%)	
**Sensory deficit before surgery (3 months)**			<0.001^a^
No sensory deficit pre-surgery	56 (93.3%)	5 (41.7%)	
Sensory deficit pre-surgery	4 (6.7%)	7 (58.3%)	
**Sens deficit before surgery (1 year)**			0.003^a^
No sensory deficit pre-surgery	34 (87.2%)	5 (45.5%)	
Sensory deficit pre-surgery	5 (12.8%)	6 (54.5%)	

a Pearson's Chi-squared test.

**Table 8:** Accumulated data of univariable analysis (pre-existing motor deficit).

	No motor deficit (N ​= ​71)	Motor deficit (N ​= ​37)	p value
**Motor deficit before surgery (discharge)**			<0.001^a^
Motor deficit pre-surgery	7 (9.9%)	33 (89.2%)	
No motor deficit pre-surgery	64 (90.1%)	4 (10.8%)	
**Motor deficit before surgery (3 months)**			< 0.001^a^
Motor deficit pre-surgery	10 (18.2%)	13 (76.5%)	
No motor deficit pre-surgery	45 (81.8%)	4 (23.5%)	
**Motor deficit before surgery (1 year)**			< 0.001^a^
Motor deficit pre-surgery	5 (14.7%)	11 (64.7%)	
No motor deficit pre-surgery	29 (85.3%)	6 (35.3%)	

a Pearson's Chi-squared test.

We did not find any statistical correlation between the age at the time of surgery, sex and operation levels, and development of medical complications (p ​> ​0.05, Pearson's Chi-squared test and Kruskal-Wallis rank sum test). Similarly, we did not find a correlation between blood and surgical time and the development of medical complications (p ​= ​0.202, 0.296, Kruskal-Wallis rank sum test).

## 4 Discussion

Many high-developed societies like USA, Germany, and Sweden encounter a growing number of patients in advanced age expanding at a high pace (Rodgers et al., 2010a; Bengtsson and Scott, 2011; Ames et al., 2016). This imposes a challenge due to described complication rates in spine surgery, which vary in the literature from 10% to almost 80% depending on the type of procedure performed (Weinstein et al., 2008; Malmivaara et al., 2007; Carreon et al., 2003; Raffo and Lauerman, 2006; Daubs et al., 2007). Daubs et al. reported 37% complications (with 20% being major complications) after posterior instrumentation for ASD (adult spinal deformity). The authors identified both age and pedicle subtraction osteotomy (PSO) as important predictors of complications (Daubs et al., 2007). Hence, most publications conclude that advanced age increases the risk for complications in spine surgery (Deyo et al., 1992, 2005; Johnsson et al., 1992; Cho et al., 2007). Posterior lumbar interbody fusion approaches, such as PLIF and TLIF, enable only implants with relatively small footprints due to the relatively small anatomical corridor created by the dural sac, exiting nerve roots, and pedicles above and below the disk space. Thus, posterior interbody fusion techniques might be associated with subsidence of the cage implanted inside the diskspace, which predisposes to an increased biomechanical load of the screws and in consequence dislodgment of the implants (Faizan et al., 2014). In contrast, anterior techniques (LLIF, ALIF, OLIF, XLIF, LTIF) are not limited by anatomical restrains and enable the insertion of implants with significantly larger footprints, which rest on a strong peripheral cortical bone on either side, hence minimizing the risk of subsidence (Faizan et al., 2014; Cappuccino et al., 2010; Malham et al., 2015; Pimenta et al., 2012; Le et al., 2012). Furthermore, the surgery can be performed minimally invasive with lesser perioperative trauma and minimal blood loss which results in reduced hospital stays, and quicker recovery time (Ozgur et al., 2006; Rodgers et al., 2010b, 2011). Therefore, theoretically LLIF, LTIF might be beneficial in the geriatric patient population. However, even though anterior approaches for interbody fusion gained popularity among spine surgeons in recent years, there are still concerns regarding risk for vascular and visceral injuries. The anterior surgery has its own learning curve; hence it requires both proficient knowledges of vascular anatomy and surgical skills to mobilize the internal organ and vessels without injury. Brau et al. reported encouraging low vascular complication rate in their large, multicenter study (1.9%) (Brau et al., 2004). A similar rate was described in two other literature reviews, with less than 5% for venous and 0.9% for arterial lacerations (Czerwein et al., 2011; Wood et al., 2010). The L4/L5 level contributes significantly to the general risk of vascular injury (Czerwein et al., 2011; Wood et al., 2010; Brau et al., 2004). Despite using minimally invasive lateral approaches to the thoracic and lumbar spine, we did not encounter any vascular or visceral complications in our material. The mean perioperative bleeding was in accordance with the available literature (Knight et al., 2009; Rodgers et al., 2010a; Karikari et al., 2011; Agarwal et al., 2018; Youssef et al., 2010). The same applied to operation time (Knight et al., 2009; Youssef et al., 2010; Isaacs et al., 2010). It is important to state that the L4/5 segment was a surgical target in only 6 (5.6%) of all cases in our study.

The lumbosacral plexus is intrinsically connected to the psoas muscle as it divides into specific nerves that descend toward limbs along the spine column (Uribe et al., 2010; Moro et al., 2003; Benglis et al., 2009). The size of the anatomical “safe corridor” that surgeons can use to reach the targeted disc space without risking injuring lumbosacral plexus within the psoas muscle decreases rapidly from almost 50% of it anterior-posterior diameter at L1/L2 to only 13.1% at L4/L5 (Regev et al., 2009). Hence, exposure to the thoracolumbar and upper lumbar levels poses different challenges (mobilization of the diaphragm, rib resection, mobilization of the lung, etc.) than exposure of lower lumbar levels. At L3/L4/L5 segments, the nerves enter the psoas and appear on its surface close to the surgical corridor. Excessive stretching and/or compressing of the plexus, during psoas manipulation and retraction can impinge the nerves and cause local ischemia, which usually leads to postoperative pain and neurological deterioration (Tohmeh et al., 2011; Youssef et al., 2010; Ochoa et al., 1971). The danger of neurological deficit is highest at L4/5 where the branches of the lumbar plexus can be found in the center of the disc (Park et al., 2010). IONM supposedly should act as a plexus detector and warn a surgeon during LLIF approaches to avoid injury of the plexus. Despite the wide use of IONM, the reported prevalence of lumbosacral plexus injury in the literature reaches 52% (Tohmeh et al., 2011; Isaacs et al., 2010; Sharma et al., 2011; Sofianos et al., 2012; Pumberger et al., 2012a, 2012b; Dakwar et al., 2010; Tormenti et al., 2010; Berjano and Lamartina, 2011). The same findings apply to postoperative dysesthesia that can occur up to 75% (Tohmeh et al., 2011; Sharma et al., 2011; Tormenti et al., 2010). Despite that IONM was not used in any of the LLIF and LTIF in our study, the rate of postoperative neurological deterioration in our cohort was much lower in comparison to the previous reports (Tohmeh et al., 2011; Isaacs et al., 2010; Sofianos et al., 2012; Pumberger et al., 2012b).

We can only speculate that a minimally invasive technique, used in all our cases, with very limited dissection contributed to the avoidance of direct injury to iliohypogastric, ilioinguinal, and lateral femoral cutaneous can be encountered in abdominal muscles, retroperitoneal space, and on the surface of the psoas. The injury of these nerves usually leads to severe post-operative thigh pain (Grunert et al., 2017; Shen et al., 2007; Anand et al., 2010). Another possible explanation of our low rate of neurological deficits is the small number of surgeries performed at L4/5 level all of which, were performed from the left side. Our surgical strategy was to gain minimally invasive access to the anterior edge of the psoas muscle following minimal and meticulous dissection, sufficient to get an insight into the anterior portion of the disk space. This plays an important role to reduce psoas retraction to a minimum (mean operation time 92 ​min).

However, we did not find any correlation between the time of surgery, level of the operated segment, blood loss and time of the surgery, and postoperative motor deficit and leg pain at discharge and follow-up except for operation time, which was close to significant at discharge and significant at 12 months for persisting sensory deficit. The only statistically significant correlation was found between preexisting status and clinical outcome at each time of follow-up control. Although the percentage of the deteriorations increased at 3 and 12 months of follow-up, so did the percentage of improved neurologic outcomes.

Spine surgery in the elderly poses an important challenge of postoperative medical complications. Depending on the type of posterior spine surgery the complication rate in the elderly varies from 18% to as high as 80% (Benz et al., 2001; Deyo et al., 1992; Weinstein et al., 2008; Carreon et al., 2003; Ahmad and Simmons, 1972). Raffo et al. reported their results after lumbar spinal fusion in geriatric patients with an overall 40% major complication rate (Raffo and Lauerman, 2006). Rodgers et al. reported a 60% complication rate for posterior instrumented spine surgery with an overall mortality rate 30% (Rodgers et al., 2010a). Daubs et al. reported 37% complications (20% being major) after posterior instrumented fusion for ASD and age was one of the predictors of complications (Daubs et al., 2007). Age, blood loss, operative time, and the number of fused levels were identified as contributing factors responsible for the high complication rate (Rodgers et al., 2010a; Carreon et al., 2003; Cho et al., 2007).

In contrast, Rodgers et al. reported a significantly lower complication rate in patients over 80 years old after MIS LLIF in comparison to PLIF (7.5% vs 30%) with a significantly lower mortality rate (2.5% vs 30%) (Rodgers et al., 2010a). Karikari et al. presented similar results with major complications of 7.4% but minor complications of 25% (Karikari et al., 2011). Agrawal et al. encountered only 9% complication but almost 2% mortality after MIS LLIF (Agarwal et al., 2018).

We did not have any mortality in the investigated group, but we encountered a high number of medical complications (36%). The gross majority (20%) were major complications. Nevertheless, it is important to highlight that 3 of the patients developed almost 30% of the total number of complications.

Similar to Rodgers et al. we did not find a contributing factor to the complication rate after anterior interbody fusion (Rodgers et al., 2011). The main difference is that the average age in our material was 76.5 years, which is 10 years older than in their publication. Several other reports did not find an association between advanced age and peri- and postoperative rate of complications in posterior, instrumented surgery but interestingly prior fusion surgery was a significant factor in the incidence of complications (Cassinelli et al., 2007; Kilincer et al., 2005; Ragab et al., 2003).

92% of all patients in our cohort were operated on with posterior decompression and fusion with an average of 5.5 levels of instrumentation before LLIF/LTIF. That also could be a possible explanation of the complication rate in our material, which is higher than described in the literature (Rodgers et al., 2010a; Karikari et al., 2011; Agarwal et al., 2018). Theoretically, the LTIF that requires manipulation with the lung and splitting of the diaphragm could be responsible for a large number of postoperative pneumonia, but we did not find any correlation between the operation level and complications. Neither did we find any correlation results between the perioperative blood loss and the time of the surgery.

Our study has several important limitations. It is important to acknowledge that it is an analysis of a heterogeneous group. Furthermore, it is based on a subgroup from a previously published study so the biases during the secondary analysis need be recognized (Krieg et al., 2019). The study also suffered an important drop-out of the patients during follow-up, which had a negative impact on statistical analysis.

## 5 Conclusion

IONM is not mandatory for LLIF procedures and neither avoids sensory nor motor deficits as presented in a larger review very recently (Nie et al., 2022). LLIF in the geriatric population has a very low risk of vascular and visceral injuries as well as neurological deterioration in postoperative follow-up. However, surgery, despite being minimally invasive was associated with a high risk of postoperative medical complications as is typical for this subgroup. LLIF/LTIF might therefore be considered in the elderly in addition to posterior instrumented surgery. Concerning the avoidance of deficits or plexopathies, awareness of the plexus anatomy inside the psoas muscle and anatomical dissection seems far more important than using undirected IONM.

## Conflict of interest

The study was financed from institutional grants. LB received teaching honoraria and research grant from Medtronic (Sweden). SK is consultants for Ulrich Medical (Ulm, Germany) and Need Inc. (Santa Monica, CA, USA); he received honoraria from Medtronic (Meerbusch, Germany), Nexstim Plc (Helsinki, Finland) and Carl Zeiss Meditec (Oberkochen, Germany). SK and BM received research grants and are consultants for Brainlab AG (Munich, Germany). BM received honoraria, consulting fees, and research grants from Medtronic (Meerbusch, Germany), icotec ag (Altstätten, Switzerland), and Relievant Medsystemy Inc., Sunnyvale, CA, USA), honoraria, and research grants from Ulrich Medical (Ulm, Germany), honoraria and consulting fees from Spineart Deutschland GmbH (Frankfurt, Germany) and DePuy Synthes (West Chester, PA, USA), and royalties from Spineart Deutschland GmbH (Frankfurt, Germany).

All authors declare that they have no conflict of interest regarding the materials used as well as the results presented in this study.
